# Supplementary figures and images for: Examining the molecular mechanisms contributing to the success of an invasive species across different ecosystems
Source: Ecol Evol. 2020 Aug 31;10(18):10254–70. doi: 10.1002/ece3.6688 (PMC7520182; doi:10.1002/ece3.6688)

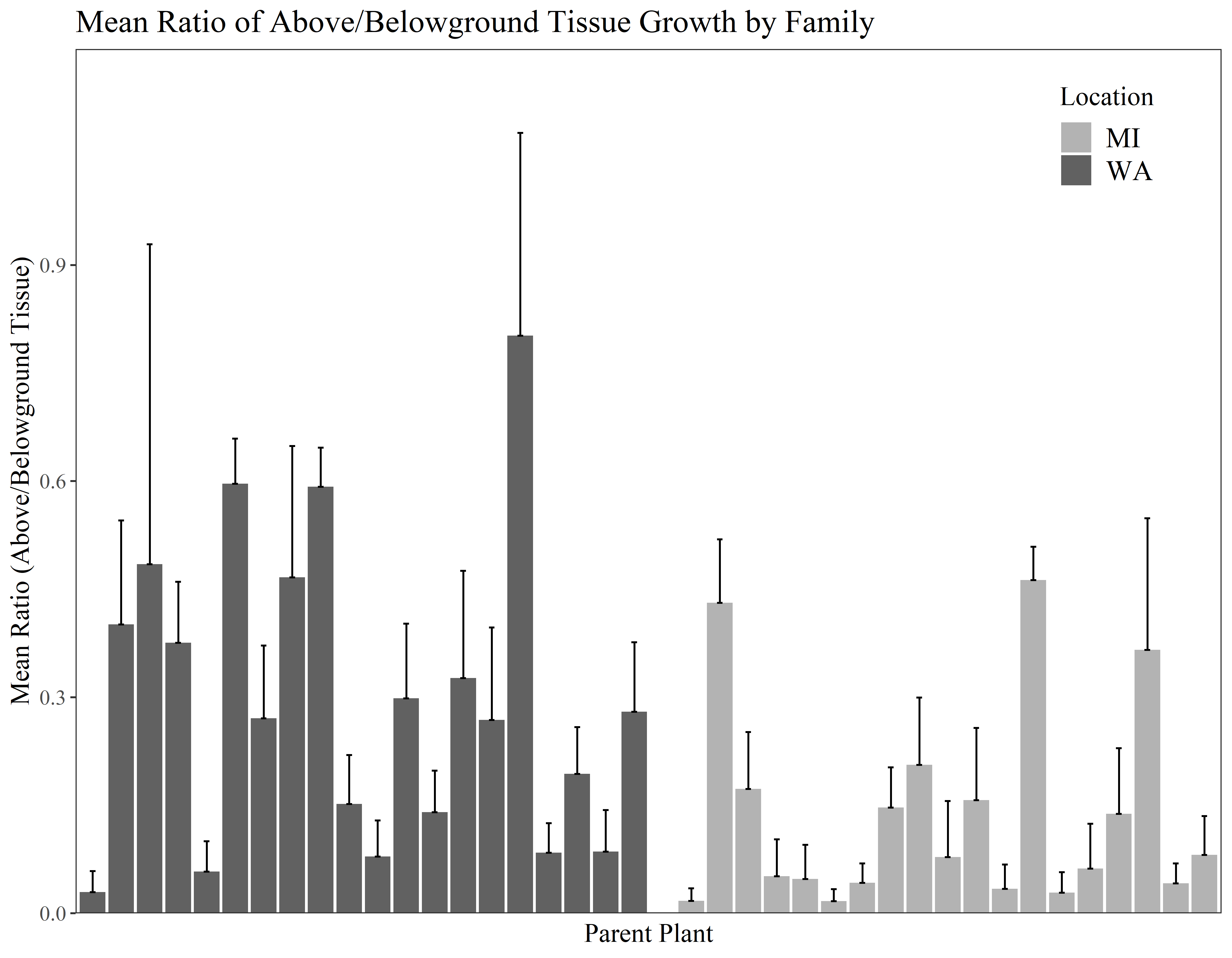

Supplement: Supplementary file 1 — Figure S1 [file ECE3-10-10254-s001.tiff]
